# Long-term drug costs per life-month gained associated with first-line treatments for unresectable or metastatic melanoma

**DOI:** 10.1186/s40164-016-0039-0

**Published:** 2016-04-11

**Authors:** Jun S. Liu, Sumati Rao

**Affiliations:** 1 Department of Statistics, Harvard University, Cambridge, MA USA; 2 Bristol-Myers Squibb, Princeton, NJ USA

**Keywords:** Ipilimumab, BRAF inhibitors, Long-term benefit, Overall survival, Drug cost

## Abstract

**Background:**

For unresectable or metastatic melanoma, first-line ipilimumab has demonstrated long-term survival benefits over a 7-year period. First-line treatment with BRAF inhibitors has demonstrated efficacy in clinical trials with up to 3 years of follow-up. The long-term comparative efficacy and costs of ipilimumab and BRAF inhibitors are unknown.

**Methods:**

Patient-level data from 12 clinical studies for ipilimumab were used. Survival data were extracted from included clinical trials for BRAF inhibitors based on a systematic literature review. Different parametric survival models, including exponential, Gompertz, log-normal, and Weibull models, were used to fit reported overall survival (OS) data and to project long-term survival for BRAF inhibitors. Survival benefits were measured in terms of total life-months gained as calculated by the area under the curve of OS Kaplan–Meier curves for the observed ipilimumab data and projected BRAF inhibitor data. Total life-months gained and cumulative costs per life-month gained were compared between ipilimumab and BRAF inhibitors.

**Results:**

The systematic literature review identified six randomized-controlled trials of BRAF inhibitors for subsequent analyses. With 7-year follow-up, ipilimumab was associated with a total of 28.5 life-months gained. Based on the Weibull model, the extrapolated total life-months gained for BRAF inhibitors were 26.5 months for dabrafenib, 21.3 months for trametinib, 14.3 months for vemurafenib, and 24.6 months for dabrafenib + trametinib. In sensitivity analyses, extrapolated total life-months gained varied across the three other models, ranging from 13.7 to 36.8 months across therapies. Cumulative costs per life-month gained with ipilimumab decreased steadily over time, while the costs remained constant for BRAF inhibitors due to continuous dosing. By year 3, cumulative costs per life-month gained were the lowest with ipilimumab; by year 7, the costs were $4281 for ipilimumab, compared with $8920 for dabrafenib, $10,211 for trametinib, $11,002 for vemurafenib, and $19,132 for the dabrafenib + trametinib combination therapy.

**Conclusions:**

Ipilimumab was associated with a better long-term cost-per-life month compared to BRAF agents. Long-term extrapolation of survival with BRAF agents was uncertain, and showed no evidence of prolonged survival compared to ipilimumab.

**Electronic supplementary material:**

The online version of this article (doi:10.1186/s40164-016-0039-0) contains supplementary material, which is available to authorized users.

## Background

Melanoma is one of the most deadly cancers, contributing to 80 % of skin cancer-related deaths [1]. It has been estimated that nearly 10,000 patients in the United States will die from melanoma in 2015 [2]. In 4 % of initial diagnoses, the cancer has already metastasized to other sites in the body [2]; in up to 40 % of early-stage melanoma, there will be disease recurrence, usually with lymph node invasion [3, 4]. Unresectable or metastatic melanoma is difficult to control because it is highly heterogeneous at the molecular level and continues to progress throughout its clinical course [5].

Treatment choices for unresectable or metastatic melanoma have rapidly evolved in the last decade due to a better understanding of cancer mechanisms [6]. Ipilimumab, dabrafenib, trametinib, vemurafenib, or dabrafenib + trametinib combination therapy are approved by the US Food and Drug Administration (FDA) as first-line treatments [7]. The immuno-oncology agent ipilimumab induces a strong anti-cancer immune response through a sustained but non-specific mechanism of activation of T cells [8–10]. In contrast, the targeted therapies dabrafenib, trametinib, and vemurafenib are directed against a specific tumor cell survival pathway, the mitogen activated protein kinase/extracellular signal-regulated kinase (MEK) pathway [11]. About 60 % of melanocytes include the mutation of a single amino acid (V600E or K) to constitutively activate the BRAF kinase, a key player in the MEK pathway [11]. Vemurafenib and dabrafenib selectively inhibit this active mutant kinase [11]; trametinib provides additional inhibition of the downstream MEK pathway. All three agents are approved for use in patients with BRAF V600E or V600K mutations; the combined treatment of dabrafenib + trametinib is associated with significantly better efficacy relative to BRAF monotherapies (e.g., dabrafenib or vemurafenib) [12–15].

Given that metastatic melanoma progresses rapidly [16, 17] and evolves unpredictably due to molecular heterogeneity [5], optimal treatment selection is crucial. Despite a wide range of available treatments, each therapy is associated with a distinct set of benefits and risks. Ipilimumab only requires a short period of treatment (four infusions over 3 months) to achieve clinical response, which can be long-lasting. In a pooled analysis of 12 clinical studies, first-line ipilimumab demonstrates long-term survival benefits over a 7-year period with survival rates plateauing and staying constant at ~21 % after 3 years [18]. However, the disadvantages of ipilimumab are that the treatment effect can be delayed by ~3 months [19] and initial response rates are low [19–24]. In contrast, BRAF inhibitors present the advantage of high initial response rates [12, 25–29]. However, response to BRAF inhibitors can be temporary, as resistance usually develops within 6 months of treatment initiation [30], and their long-term efficacy remains unclear. A recent study among patients treated with vemurafenib shows that 3- and 4-year survival rates are 26 and 19 %, respectively [31]. However, for other BRAF treatments, maximum follow-up periods in clinical trials are only about 2–3 years. In addition, for a large proportion of patients with melanoma—those who have wild-type BRAF—treatment with BRAF inhibitors is not approved by the FDA due to the possibility of disease exacerbation [32].

The high costs of cancer medicines are increasingly drawing public attention. Costs, together with clinical benefit (i.e., efficacy) and toxicity, are included in the American Society of Clinical Oncology (ASCO) conceptual framework to assess the value of cancer treatments [33]. To date, the long-term comparative value, including efficacy and costs, of first-line treatments with ipilimumab and BRAF inhibitors remains unknown. The current study aimed to address this knowledge gap by comparing long-term survival and costs of ipilimumab, dabrafenib, trametinib, vemurafenib, and dabrafenib + trametinib combination therapy. A systematic literature review was conducted to identify randomized controlled trials (RCTs) of BRAF inhibitors as first-line treatments for unresectable or metastatic melanoma. Based on the reported overall survival results of these treatments, parametric survival models were used to project long-term survival benefits, measured in total life-months gained. Furthermore, in order to fairly represent long-term drug costs for ipilimumab with limited doses versus BRAF inhibitors with a continuous dosing schedule, the cumulative drug costs per life-month gained were calculated and compared descriptively.

## Results

### Systematic literature review

**Fig. 1:**
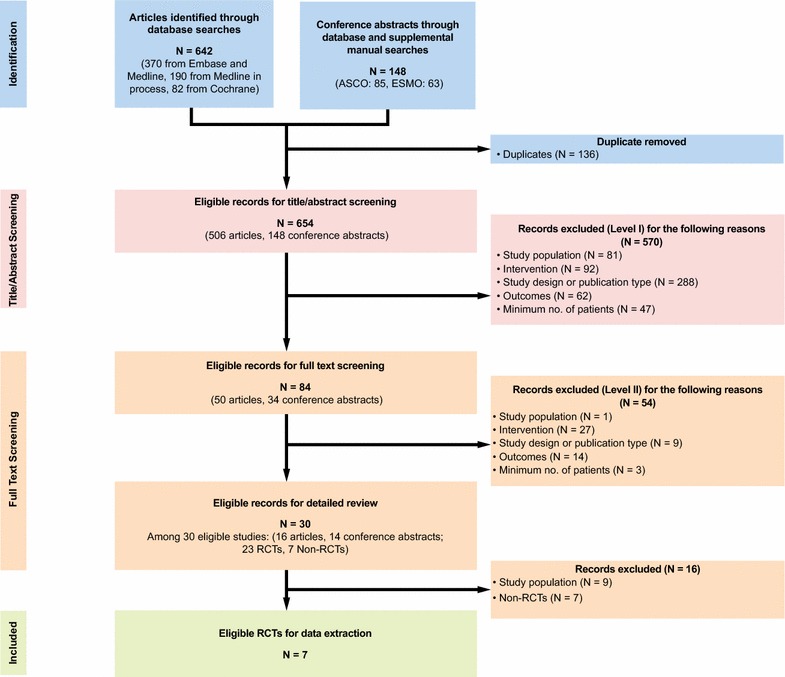
PRISMA diagram for systematic literature review

A total of 654 studies were initially identified from database searches and supplemental manual searches of recent ASCO and European Society for Medical Oncology (ESMO) conference abstracts (Fig. 1). After 2 levels of screening, 30 publications were then evaluated in detail for their eligibility for the study’s analysis; among these, seven were excluded because they were not RCTs, nine were excluded because they reported second-line or mixed first- and second-line treatments (except for trametinib, of which the only available RCT METRIC trial [34] included mixed first- and second-line patients), seven were excluded because they were publications of the same trial, and only the latest results were included for subsequent analysis.

A total of six RCTs investigating BRAF inhibitors for previously untreated metastatic melanoma reported overall survival results and were included in this study: dabrafenib was investigated in trials BREAK-3 [35, 36] and COMBI-d [13, 37]; trametinib was investigated in trial METRIC [34]; vemurafenib was investigated in trials BRIM3 [26, 28], COMBI-v [12], and co-BRIM [38]; and dabrafenib + trametinib was investigated in trials COMBI-d [13, 37] and COMBI-v [12]. Among BRAF inhibitors, median overall survival ranged from 9.2 months (for vemurafenib) [28] to 18.3 months (for dabrafenib + trametinib) [12]. Median follow-up length ranged from 3.8 months (for vemurafenib) [28] to 24 months (for dabrafenib + trametinib) [37].

### Parametric survival modeling for overall survival following first-line BRAF inhibitors

Four parametric survival models (Weibull, exponential, Gompertz, and log-normal) were used to fit the reported overall survival results of BRAF inhibitors. The Akaike information criterion (AIC) value, an indicator of a model’s goodness-of-fit, did not differ substantially across the four models for each BRAF inhibitor (Additional file 1: Table S1). There was no specific model that had the best fit for all BRAF inhibitors. The Weibull model had been previously used to analyze overall survival of patients with metastatic melanoma [39, 40], so it was chosen as the base-case model. The other three models were used as sensitivity analyses.

**Table 1:** Life-months gained

Melanoma treatment	Life-months over all available follow-up	Projected life-months for all drugs up to 7 years^b^			
	Life-months^a^	Weibull	Exponential	Gompertz	Log-normal
Ipilimumab	28.52	N/A	N/A	N/A	N/A
Dabrafenib	21.47	26.51^c^	30.03	26.50	30.52
Trametinib	7.32	21.25^c^	26.53	16.94	36.69
Vemurafenib	13.00	14.28^c^	20.65	13.69	18.10
Dabrafenib + trametinib	16.78	24.55^c^	36.75	21.88	33.12

^a^Life-months were calculated by the area under the curve of the Kaplan–Meier curves of overall survival for different drugs

^b^Parametric survival modeling was used to extrapolate the survival probabilities of dabrafenib, trametinib, vemurafenib, and dabrafenib + trametinib. Weibull model was used as the primary model (results denoted with ^c^). Other parametric models (exponential, Gompertz, and log-normal) were used as sensitivity analysis

### Total life-months gained

**Fig. 2:**
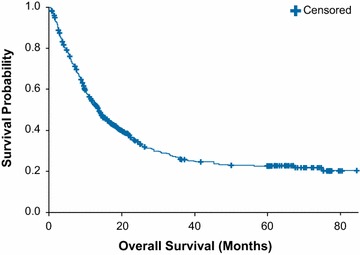
First-line ipilimumab overall survival for up to 7 years based on Schadendorf et al. 18

Survival benefits were measured by the area under the curve of the overall survival Kaplan–Meier curves as total life-months gained for the observed ipilimumab data and the projected BRAF inhibitor data. The Kaplan–Meier curve of observed long-term overall survival following first-line ipilimumab based on patient-level data from a pooled analysis of 12 clinical studies [18] is shown in Fig. 2. With 7-year follow-up, ipilimumab was associated with a total of 28.5 life-months gained per patient. For BRAF inhibitors, which have relatively short follow-up, the total observed life-months gained per patient ranged from 7.3 months (for trametinib) to 21.5 months (for dabrafenib) (Table 1).

**Fig. 3:**
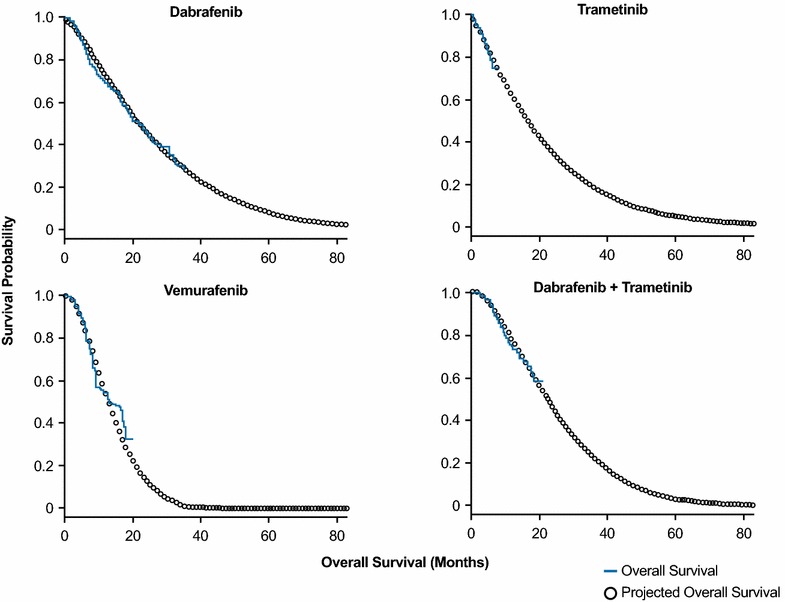
Projected overall survival for up to 7 years following first-line BRAF inhibitors based on Weibull model. The *solid line* indicates observed overall survival and the line of *circles* indicates projected overall survival based on parametric survival modeling

The Kaplan–Meier curves of observed and projected long-term overall survival following first-line treatment with BRAF inhibitors are shown in Fig. 3 for the base-case Weibull model. With projected 7-year follow-up, the extrapolated total life-months gained per patient for BRAF inhibitors were 26.5 months for dabrafenib, 21.3 months for trametinib, 14.3 months for vemurafenib, and 24.6 months for dabrafenib + trametinib, based on the Weibull model (Table 1). In sensitivity analyses, extrapolated total life-months varied across three other models, ranging from a low of 13.7 months (for vemurafenib) to a high of 36.8 months (for dabrafenib + trametinib) across therapies (Table 1).

**Fig. 4:**
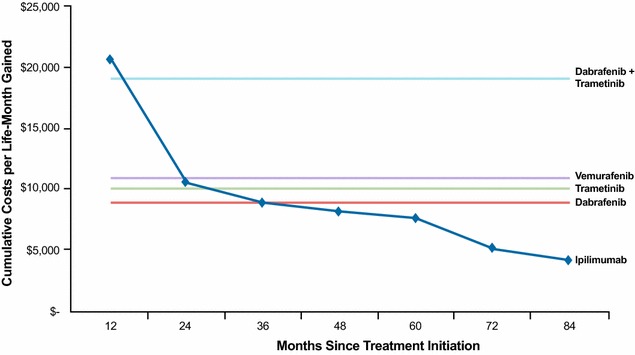
Cumulative costs per life-month gained. *1* Drug unit costs (as of March 2015) were based on the WAC from RED BOOK online^®^. *2* First-line ipilimumab long-term overall survival and average patient weight (78.7 kg) were based on individual patient data used in Schadendorf et al. [18]. Drug cost for 10 mg/kg dose was assumed to be the same as 3 mg/kg. The total cost of ipilimumab (1 dose every 3 weeks, 4 doses in total) was incurred during the first half year since drug initiation

### Cumulative costs per life-month gained

The cumulative costs per life-month gained with BRAF inhibitors remained constant over time, as patients are dosed chronically with these treatments. In contrast, ipilimumab is administered with fixed doses, four infusions over 3 months. Therefore the cumulative costs per life-month gained with ipilimumab decreased steadily over time (Fig. 4). By year 3, the cumulative costs per life-month gained were lower for ipilimumab relative to all BRAF inhibitors. By year 7, cumulative costs per life-month gained were $4281 for ipilimumab, $8920 for dabrafenib, $10,211 for trametinib, $11,002 for vemurafenib, and $19,132 for dabrafenib + trametinib.

## Discussion

Among the approved first-line treatments for unresectable or metastatic melanoma, there has not been any head-to-head RCT comparing ipilimumab, dabrafenib, trametinib, vemurafenib, and dabrafenib + trametinib. Ipilimumab is associated with an established long-term survival benefit [18], whereas BRAF inhibitors are associated with high initial response [12–14, 25–29, 41, 42], but with limited long-term data. To provide additional evidence on the comparative value between ipilimumab and BRAF inhibitors, this study was undertaken to assess the long-term survival benefit and costs of these agents.

For the current study, parametric survival models were used to project long-term overall survival for first-line treatment with BRAF inhibitors, as the reported follow-up periods for these agents (~3 years) are much shorter compared with that reported for ipilimumab (~7 years) [12, 13, 18, 26, 28, 34–38]. In the current study, among all four models used, the Weibull model provided a good fit of overall survival data for all four BRAF inhibitors. The Weibull model has also been previously used to build prognostic models for overall survival among patients with metastatic melanoma [39, 40].

Using the Weibull model as the base-case, the current study showed that ipilimumab was associated with the highest total life-months gained (28.5 months) relative to all BRAF inhibitors (ranging from 14.3 to 26.5 months) for the same 7-year follow-up period. Sensitivity analyses using the other three parametric survival models led to a range of possible extrapolated total life-months gained for BRAF inhibitors. In these projections, BRAF inhibitors sometimes, but never consistently, outperformed ipilimumab. Overall, combining base-case and sensitivity analysis results, this study showed no differences in terms of long-term survival between BRAF inhibitors compared to ipilimumab for the first-line treatment of metastatic melanoma.

Ipilimumab is administered in a fixed number of doses (4 doses over 3 months) and thus the drug costs are fixed, whereas BRAF inhibitors are dosed chronically and thus the drug costs are dependent upon the duration of treatment. To provide a fair comparison of long-term costs, cumulative drug costs per life-month gained were compared between ipilimumab and BRAF inhibitors. Such costs for BRAF inhibitors were constant and equal to the monthly cost of these treatments because by definition, total life-months gained included only patients alive and continuing treatment. The results showed that ipilimumab was associated with higher short-term cumulative costs per life-month gained (e.g., years 1 and 2) compared with BRAF inhibitors, but the costs of ipilimumab per life-year gained continued to decline over time and by year 3 were below those of BRAF inhibitors. With all available 7-year follow-up, ipilimumab was associated with cumulative costs per life-month gained of $4281, which was ~50 to 60 % less than those of BRAF monotherapies and ~80 % less than those of dabrafenib + trametinib combination therapy. These calculations are based on the assumption that patients treated with ipilimumab receive all four doses, when in practice approximately 78 % of patients receive the full, 4-dose regimen [43]. Thus, ipilimumab’s costs per life-month gained results are conservative.

The findings of this study have important implications for healthcare decision makers. Despite initial lower response rates, an immune-oncology agent with established long-term efficacy like ipilimumab can provide a lasting survival benefit for a subset of patients and result in lower costs overall. These are important considerations for third-party payers in particular, who are faced with rapidly-increasing healthcare expenditures.

There are several limitations with the current study. First, sensitivity analyses of parametric survival modeling of overall survival showed that estimated total life-months gained by BRAF inhibitors were sensitive to the specific model used. Therefore, there were uncertainties with current findings, so they should be interpreted with caution. Second, trametinib data included mixed first- and second-line treatments, as the METRIC trial [34] was the only RCT for trametinib. Thus, survival results for trametinib used in this study might be underestimated. Third, the current analysis used first-line ipilimumab data from a pooled analysis of 12 clinical studies [18], which included patients treated with the on-label dose of 3 mg/kg, as well as other experimental doses (0.1, 0.3, 1, 2, 5, 9, and 10 mg/kg). However, we did not expect the use of various doses to significantly affect our findings, as Schadendorf et al. 2015 noted similar efficacy with all doses [18]. Fourth, in the calculation of drug costs per life-month gained, only the wholesale acquisition costs (WAC) of drugs were used. Other important cost components, such as drug administration costs, patient out-of-pocket costs, and costs for managing adverse events, were not considered. Fifth, BRAF mutation status was not available in the patient-level data of ipilimumab. Thus, the results for ipilimumab were based on all patients with metastatic melanoma, irrespective of BRAF mutation status, whereas the results for BRAF inhibitors were based on patients with BRAF mutation only. Sixth, many of the included trials did not have control arms given the agents’ effectiveness in preclinical and early clinical testing. In addition, there was a high degree of heterogeneity among the studies with control arms in terms of chemotherapies administered. Thus, a chemotherapy control could not be incorporated into the current study, and the life-months gained for all drugs are relative to no treatment (and not relative to standard of care/chemotherapy). Adding a chemotherapy control would reduce the estimated life-months gained and likely lower the costs per life-months gained for all treatments. Finally, at the time of study, newer programmed cell death protein-1 (PD-1)-targeting agents nivolumab and pembrolizumab had not been approved for first-line use and thus they were not included in the current analyses. Currently, the National Comprehensive Cancer Network (NCCN) guidelines recommend nivolumab (category 1) and pembrolizumab as systemic, first-line therapies for metastatic or unresectable melanoma [7]. The UK National Institute for Health and Care Excellence (NICE) has recommended nivolumab as an option for treating advanced melanoma, and pembrolizumab as a second-line option for treating advanced melanoma following disease progression with ipilimumab (or a first-line option when a discount is provided) [44, 45]. In addition, nivolumab + ipilimumab combination therapy has recently been granted an accelerated approval as a treatment for BRAF V600 wild-type unresectable or metastatic melanoma in the US based on findings from the CheckMate-069 [24, 46]. Future studies on PD-1 agents are needed.

## Conclusions

Ipilimumab was associated with a better long-term cost-per-life month compared with BRAF inhibitors. Long-term extrapolation of survival with BRAF inhibitors was uncertain and showed no evidence of prolonged survival compared with ipilimumab.

## Methods

### Data sources

For ipilimumab, patient-level data from 12 clinical studies (MDX010-20, CA184024, CA184022, CA184008, CA184007, CA184004, CA184042, CA184332, CA184338, NCI04C0083, and NCI03C0109) was used. These studies were described in detail in Schadendorf et al. 2015 [18]. The current analysis focused specifically on first-line use of ipilimumab. Drug costs were based on WAC from RED BOOK Online^®^ (as of April 2015). Reported overall survival results for BRAF inhibitors were systematically reviewed and extracted from published RCTs.

### Systematic literature review

A systematic literature review of clinical trials of BRAF inhibitors was performed on August 18, 2014. The databases included in this search were: MEDLINE (Inception—August 18, 2014), MEDLINE In-Process (up to August 18, 2014), EMBASE (Inception—August 18, 2014), and the Cochrane Central Register of Controlled Trials (Inception—August 18, 2014). In addition, conference proceedings from 2012–2014 ASCO and ESMO annual conferences were manually searched to supplement the database search results. During the search process, two researchers reviewed all the citations independently. Any disagreements were resolved by discussion with, or independent arbitration by, a third reviewer. In order to be included for this study, eligible publications had to (1) have been conducted among adult patients with unresectable or metastatic (stage IIIc or IV) melanoma, (2) include phase II/III clinical trials of at least 50 patients, (3) include first-line treatment of dabrafenib, trametinib, vemurafenib, or trametinib + dabrafenib, and (4) include one of the following efficacy outcomes: OS, progression-free survival (PFS) or time to progression (TTP), or response rates. For included studies, relevant data were extracted into a data collection spreadsheet with prepared fields.

### Statistical analyses

Total life-months gained were calculated as the area under the curve of the Kaplan–Meier curve of overall survival (the sum of time multiplied by the proportion of patients who are alive at that time) for each agent. First, total life-months gained for all drugs were calculated based on the observed overall survival for available follow-up periods. Then, total life-months gained for BRAF inhibitors were calculated based on the extrapolated overall survival from parametric survival modeling. The cumulative costs per life-month gained were calculated by dividing the cumulative total drug costs by the total life-months gained for each drug. Cumulative total drug costs were calculated based on WAC and on-label dosing.

Kaplan–Meier analysis was conducted using patient-level data of first-line ipilimumab. For BRAF inhibitors, data from published overall survival Kaplan–Meier curves of included studies were extracted using Engauge digitization software to create “pseudo” patient-level data using methodology recommended by NICE [47]. Different parametric survival models, including exponential, Gompertz, log-normal, and Weibull models, were used to fit the “pseudo” patient-level data and subsequently project long-term OS. The AIC values of different models were compared to help select the model for base-case and sensitivity analyses. The area under the curve of the observed and projected overall survival Kaplan–Meier curves were calculated to derive the total life-months gained. Both total life-months gained and cumulative costs per life-month gained were compared descriptively between ipilimumab and BRAF inhibitors. All analyses were conducted in R statistical software.

## Additional file

**Additional file 1: Table S1. **Goodness-of-fit statistics of parametric survival models for overall survival data of BRAF inhibitors.

## Abbreviations

AIC: Akaike information criterion
ASCO: American Society of Clinical Oncology
AUC: area under the curve
ESMO: European Society for Medical Oncology
MEK: mitogen activated protein kinase/extracellular signal-regulated kinase
NICE: National Institute for Health and Care Excellence
OS: overall survival
PFS: progression-free survival
TTP: time to progression
RR: response rate
WAC: wholesale acquisition cost
