# Supplementary material for: Long-term drug costs per life-month gained associated with first-line treatments for unresectable or metastatic melanoma
Source: Exp Hematol Oncol. 2016 Apr 11;5:9. doi: 10.1186/s40164-016-0039-0 (PMC4827222; doi:10.1186/s40164-016-0039-0)
Supplement: Supplementary file 1 — Additional file 1: Table S1. Goodness-of-fit statistics of parametric survival models for overall survival data of BRAF inhibitors. [file 40164_2016_39_MOESM1_ESM.docx]

# Supplemental Materials

## Supplemental Table 1. Goodness-of-fit statistics of parametric survival models for overall survival data of BRAF inhibitors

|  | **Parametric model AIC** | | | |
| --- | --- | --- | --- | --- |
| **BRAF agent** | **Exponential** | **Weibull** | **Lognormal** | **Gompertz** |
| Dabrafenib | 1535.001 | 1524.428 | 1507.669 | 1533.978 |
| Dabrafenib + trametinib | 1336.514 | 1300.701 | 1294.282 | 1320.188 |
| Trametinib | 349.6516 | 350.5827 | 351.6243 | 351.2334 |
| Vemurafenib | 3036.793 | 2908.342 | 2894.478 | 2970.726 |
